# A Novel Approach for Implant Rehabilitation Combined with Immediate Bone and Soft-Tissue Augmentation in a Compromised Socket—A B2S Approach: Case Report with a 2-Year Follow-Up

**DOI:** 10.1155/2023/1376588

**Published:** 2023-03-28

**Authors:** Baruch S. Bernatskiy, Algirdas Puišys

**Affiliations:** ^1^Private Practice, Queen Nella Clinic, Tel Aviv, Israel; ^2^Private Practice, VIC Clinic, Vivulskio 7, Vilnius LT 01362, Lithuania

## Abstract

In this case report, we aimed to describe a novel approach for aesthetic rehabilitation of the anterior maxilla that combined immediate implant installation with the “Bone2Soft Tissue Reconstruction” (B2S technique), which involves the use of a triple graft harvested from the maxillary tuberosity. The regeneration potential of a tuberosity graft appeared to surpass that of corticocancellous bone grafts harvested from other intraoral donor sites and allowed for quicker regeneration of both bone and soft tissue. The B2S technique extended the indications for immediate implant placement and ridge augmentation to cases involving severe bone resorption and other complex clinical scenarios. Owing to the good visualization facilitated by open-flap access, the surgical procedures can be completed in a single intervention, which will be beneficial for both doctors and patients.

## 1. Introduction

Aesthetic rehabilitation with dental implants requires hard- and soft-tissue augmentation either prior to or simultaneously with implant installation depending on the complexity of the defect. Guided bone regeneration (GBR) is one of the most documented bone-augmentation techniques. The use of a particulate inorganic xenogenic bone graft combined with a resorbable barrier membrane allows for significant bone formation both horizontally and vertically [1–3]. Moreover, most studies have confirmed that implant survival rates are very high when implant placement is combined with GBR, and they recommended immediate or early implant placement to ensure crestal bone stability [4, 5]. Simultaneously, some authors have highlighted the lack of homogeneity in studies and protocols since there are too many clinical variables to consider [6].

There is sufficient evidence indicating that immediate implant surgery performed simultaneously with GBR is efficient in cases involving minor or moderate horizontal defects (fenestration or dehiscence), in which an implant is placed within bony housing. However, in cases involving a vertical defect, the interval between GBR and implant surgery should be 9–13 months [7]. Such an extended waiting period may not be acceptable when the defect is located within the aesthetic area, necessitating an ongoing search for novel approaches [8].

Recently, a distinct tendency has been observed in GBR procedures involving particulate bovine bone minerals and native non-crosslinked membranes, which is the gold standard technique for bone augmentation. Most clinicians have begun to use a 50% : 50% mixture containing autogenous bone particles [9]. Simultaneously, some authors have acknowledged that autogenous bone blocks are superior to autogenous bone particles in terms of the amount of bone fill [10].

In this case report, we aimed to describe the reconstruction of a severe bone defect in the anterior maxilla, which was augmented with a tuberosity triple graft simultaneously with implant placement during a single surgical intervention. The use of tuberosity grafts has been described in only a few articles in the literature. Therefore, we considered an evaluation of the behavior of autogenous grafts as alternative materials for bone augmentation, which could lead to better outcomes in compromised cases, to be of clinical interest.

## 2. Case Presentation

### 2.1. Extra- and Intraoral Examinations

**Figure 1:**
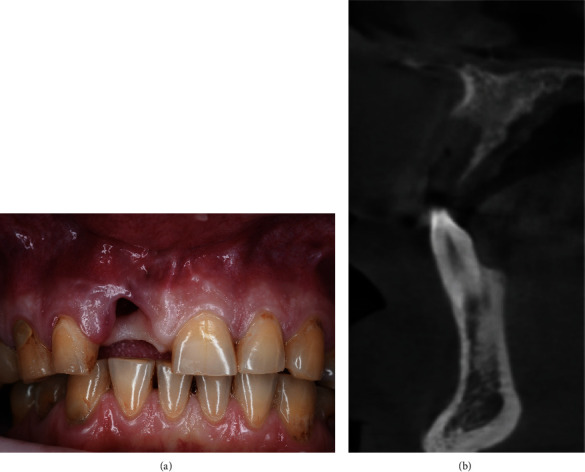
Baseline status.

**Figure 2:**
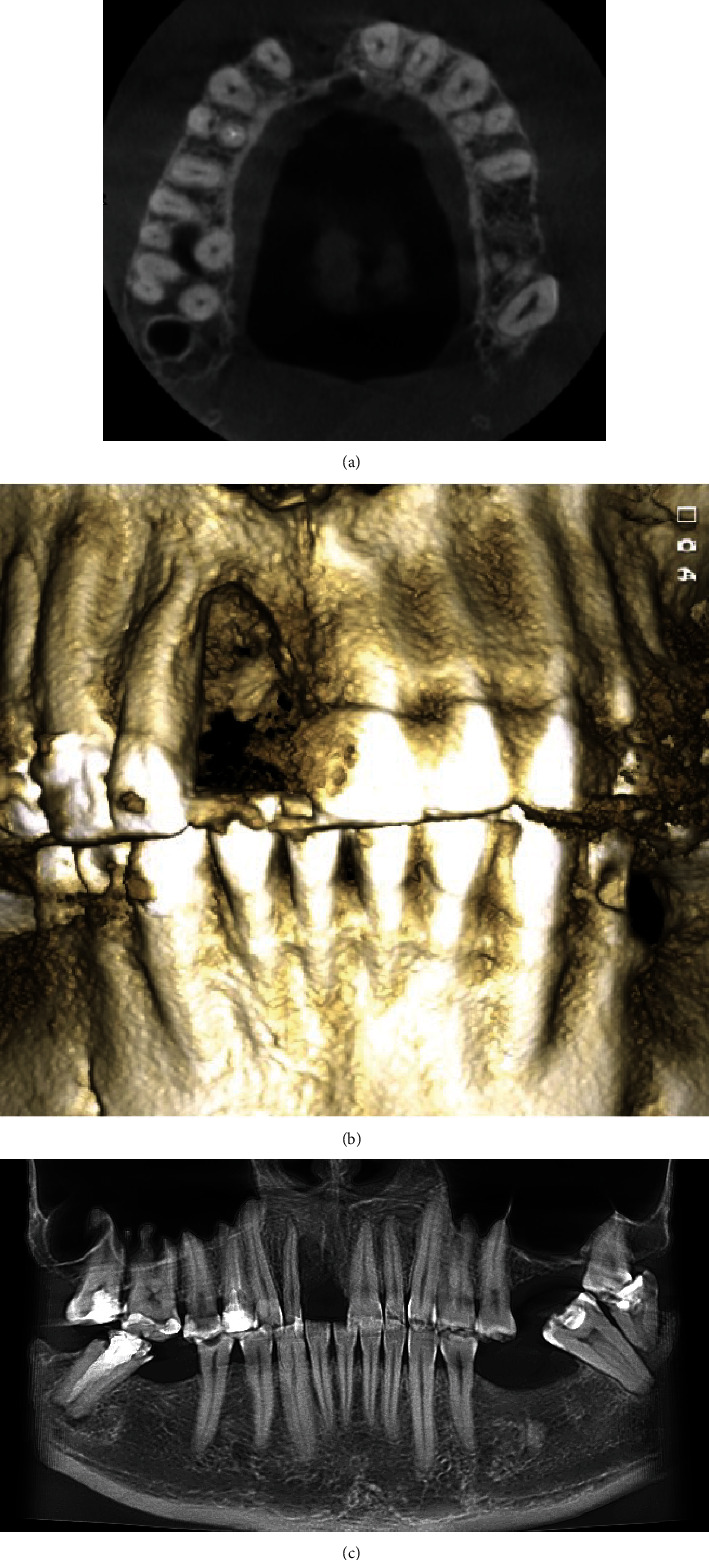
Cone-beam computed tomography scans obtained before the surgery.

A 48-year-old man, who was a heavy smoker, presented to the clinic with a missing right central incisor (Figures 1(a) and 1(b)). Dental anamnesis revealed that the tooth had been extracted 3 weeks ago. A preoperative extraoral examination revealed a moderate lip line. An intraoral examination of the site revealed gingival recession along with a lack of keratinized tissue, whereas the mesial and distal papillae were well-shaped and keratinized. The thick soft-tissue phenotype at the neighboring sites could be explained by the patient's heavy smoking. A radiographic examination confirmed the complete absence of a buccal cortical plate up to the former apex and a missing interproximal peak between the central and lateral incisors, as well as 1.5 mm of marginal bone resorption palatally (Figures 2(a), 2(b), and 2(c)). A comprehensive intraoral evaluation indicated an abundance of abfractions, which may have been the result of traumatic brushing, gastroesophageal reflux disease, or a combination of the two, as well as wear facets indicating occlusion stability-related problems and bruxing activities.

### 2.2. Treatment Objectives

The treatment goal was to restore function and aesthetics by replacing the missing tooth with a dental implant and augmenting the missing bone and soft tissue. The patient was very keen on shortening the treatment time; therefore, the procedures were performed in a single surgical intervention that combined implant placement and alveolar ridge augmentation.

### 2.3. Treatment

**Figure 3:**
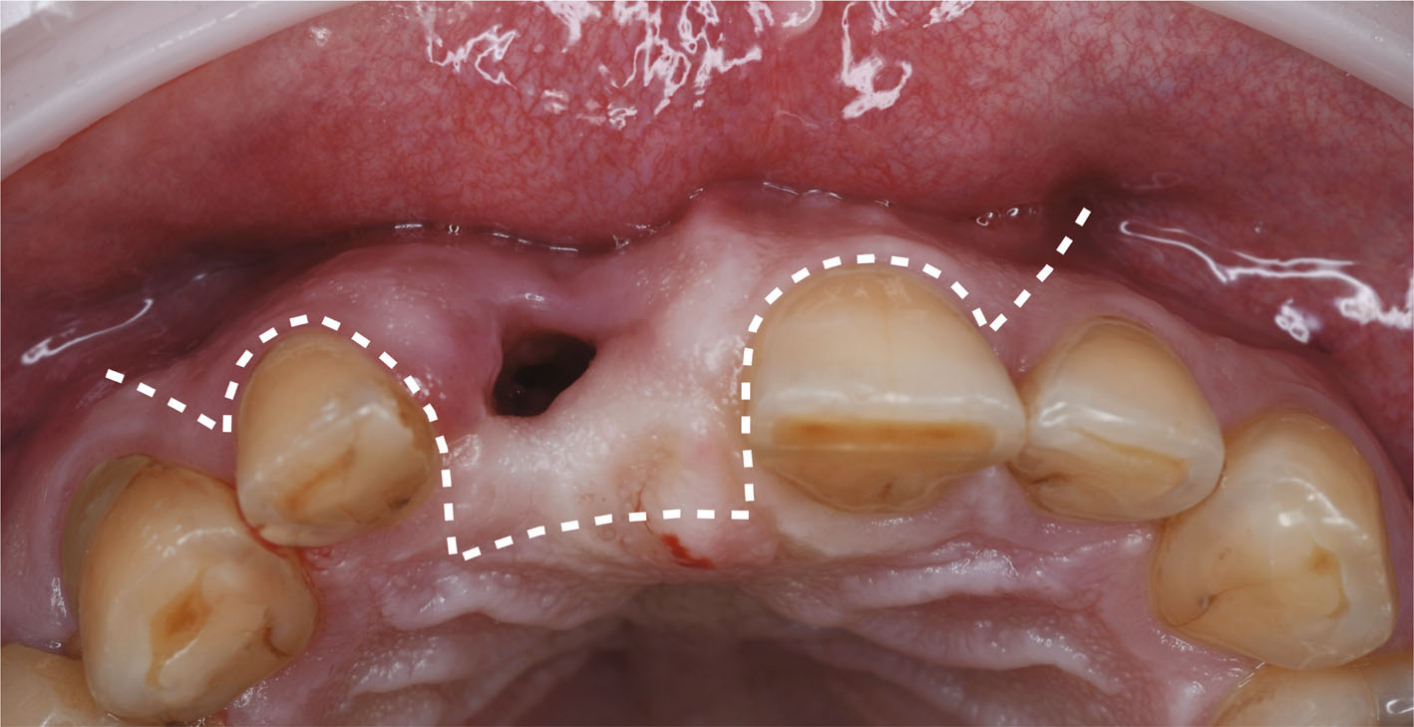
Flap design.

**Figure 4:**
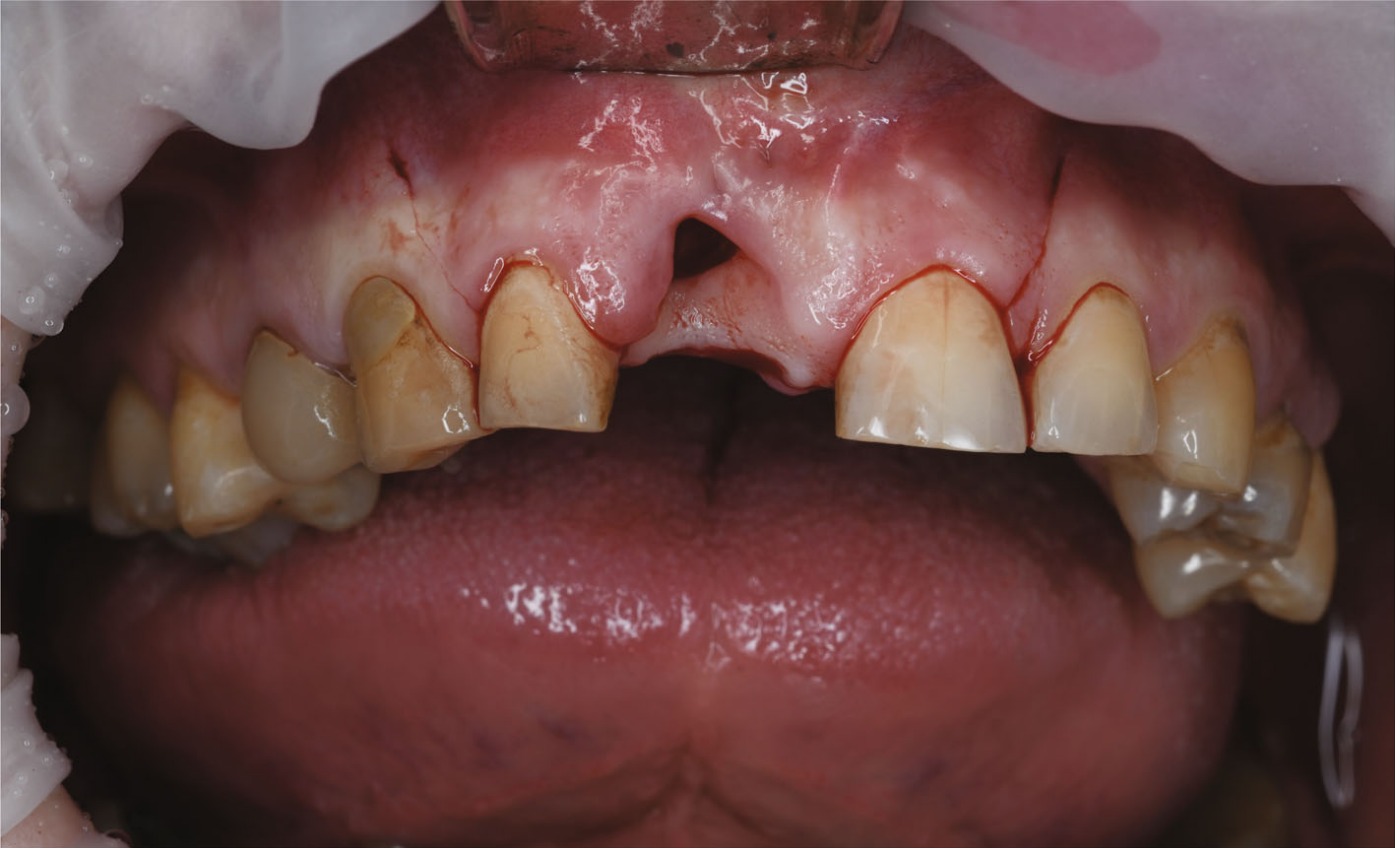
Vertical hockey-stick-shaped releasing incisions.

A systemic antibiotic, amoxicillin (875 mg), was administered along with clavulanic acid (125 mg) 1 hour prior to the surgery; subsequently, the patient continued to receive the antibiotic treatment for 7 days twice daily. Local infiltration anesthesia was induced using Ubistesin Forte 4%, articaine hydrochloride 4%, and adrenaline (epinephrine) 1 : 100,000 (3M Deutschland GmbH, Neuss, Germany). The incision was made using a 15C blade (10-256-15; Hu-Friedy Mfg. Co., LLC, Frankfurt am Main, Germany) as follows. The crestal incision was shifted 3 mm palatally to facilitate suturing and wound closure, as well as to move away from the augmented area (Figure 3). Intrasulcular incisions around the neighboring teeth were made only buccally and extended as far as the distal line angles of a tooth located remotely from the defect. Vertical releasing incisions were started from the gingival margin and were advanced as deep as the mucogingival border with one stroke in a hockey-stick shape (Figure 4). Vertical incisions were made at a 90° angle to the bone, and a new blade was used each time contact was made with the bone.

**Figure 5:**
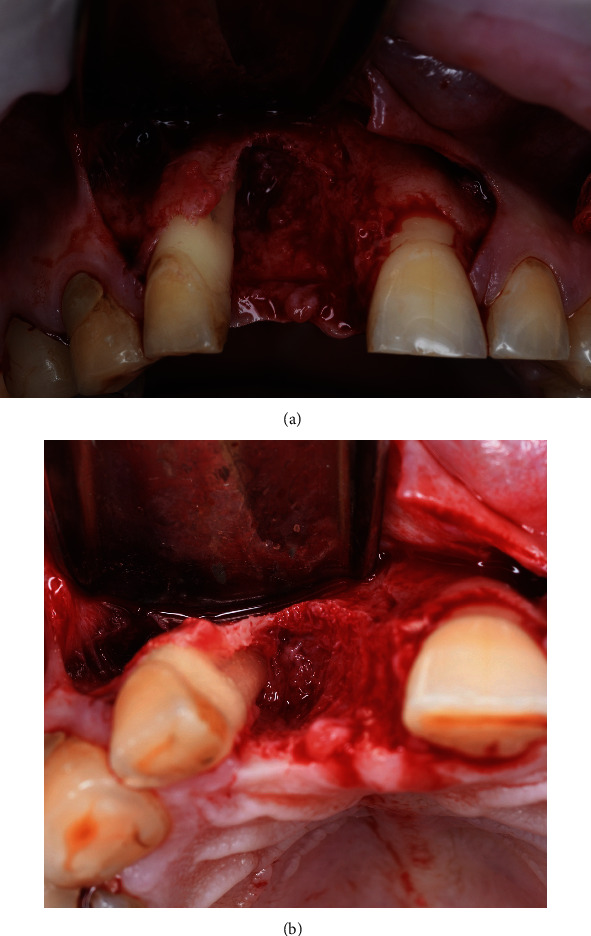
Defect topography.

In order to access and visualize the defect, a full-thickness flap was reflected (Figures 5(a) and 5(b)) using a microsurgical periosteal elevator, also called “Mini Me”, which was developed by Istvan Urban, in areas with thinner tissues (PFIWDS1MKX; Hu-Friedy Mfg. Co. LLC, Frankfurt am Main, Germany) and a Prichard periosteal elevator in areas with thicker soft tissues (PPR3; Hu-Friedy Mfg. Co. LLC, Frankfurt am Main, Germany). Granulation tissues were thoroughly removed from the sockets using a Lucas surgical curette (CL86; Hu-Friedy Mfg. Co. LLC, Frankfurt am Main, Germany).

**Figure 6:**
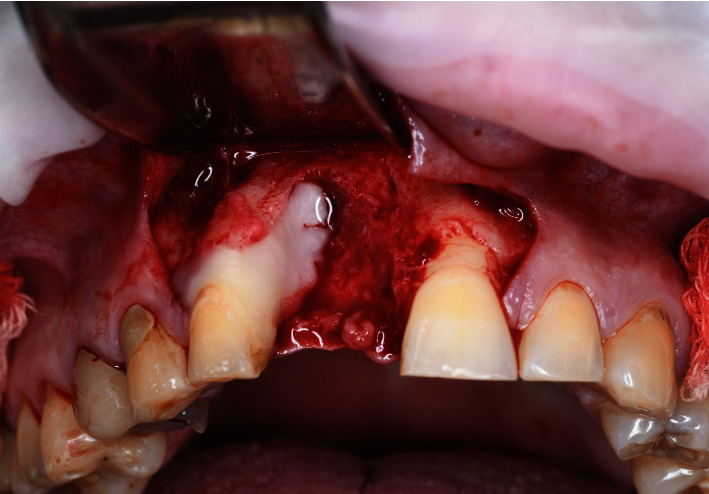
Root conditioning with ethylenediaminetetraacetic acid and enamel matrix derivative application.

The first step was scaling and root planing of the lateral incisor located next to the defect, followed by 24% Ethylenediamide tetraacetic acid (EDTA) application for decontaminating the surface; subsequently, an enamel matrix derivative (Emdogain®/PrefGel®, Straumann Holding AG, Basel, Switzerland) was applied to promote cementogenesis and periodontal regeneration (Figure 6).

The standard AstraTech TX protocol was used to prepare the implant bed (Dentsply Sirona; Dentsply Implants Manufacturing GmbH, Hanau, Germany). The process was started with a 2.0 mm pilot drill applied in the projection of the future crown. Drilling was first performed to mark the vertical position of the implant neck (2 mm apical to the palatal wall), after which the drill was placed almost perpendicular to the marginal bone to create a small notch to avoid further drill slipping and deviation. Next, the pilot drill was vertically aligned along the future implant axis and reached a full implant depth of 11 mm. Subsequently, drilling was performed along the palatal bone wall.

**Figure 7:**
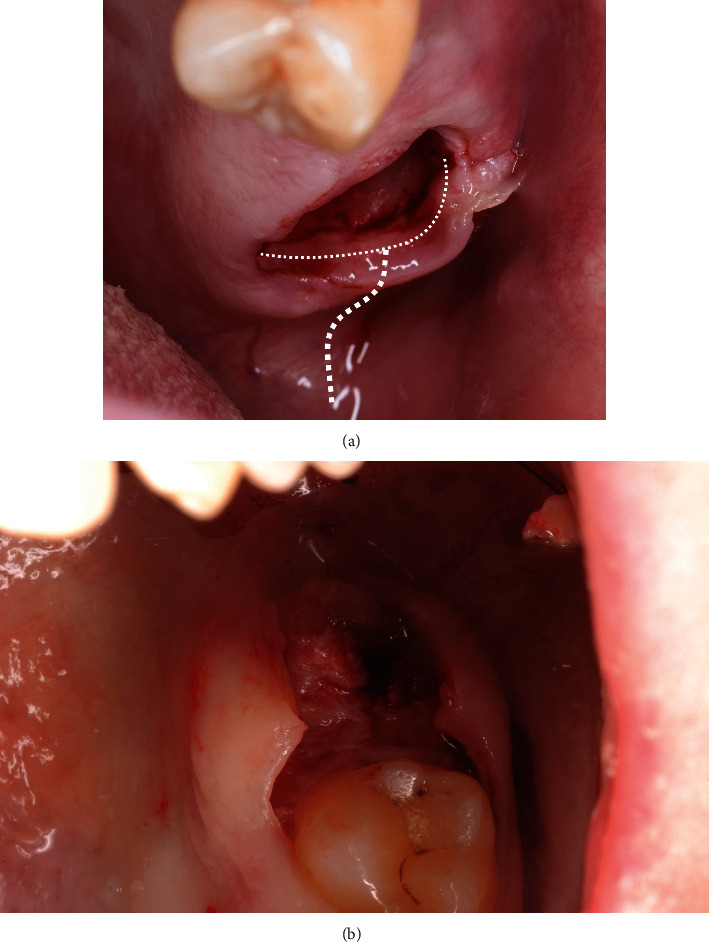
(a) Split-thickness incision to facilitate access for the chisel. (b) Split-thickness incision to facilitate access for the chisel.

After implant bed preparation, a graft was harvested to fill in the alveolar defect, which was first carefully measured with a periodontal probe (PCP UNC 156; Hu-Friedy Mfg. Co. LLC, Frankfurt am Main, Germany) to calculate the graft size. The maxillary tuberosity was used as the donor site for harvesting the bone block for augmentation. To gain access to the tuberosity, we performed extraction of the third molar within the same quadrant, which had been discussed with and approved by the patient prior to the surgery (Figures 7(a) and 7(b)).

The starting point of the crestal incision was moved distally to the pterygomandibular raphe; the incision was then continued with one stroke mesially toward the socket margin by using a number 12 blade (10-256-12; Hu-Friedy Mfg. Co. LLC, Frankfurt am Main, Germany), which divided the tuber into two equal parts. The buccal and lingual flaps were then split using a new 15C blade (10-256-15; Hu-Friedy Mfg. Co. LLC, Frankfurt am Main, Germany) and were then elevated, leaving the periosteum and submucosal layers on the tuber. This incision allowed for the visualization and control of the soft-tissue layer over the tuberosity, which was important for further augmentation. To prepare for graft harvesting, another splitting incision was made from inside the distal border of the socket at a 2 mm distance from the gingival margin parallel to the occlusal surface by using a new 15C blade (10-256-15; Hu-Friedy Mfg. Co. LLC, Frankfurt am Main, Germany).

**Figure 8:**
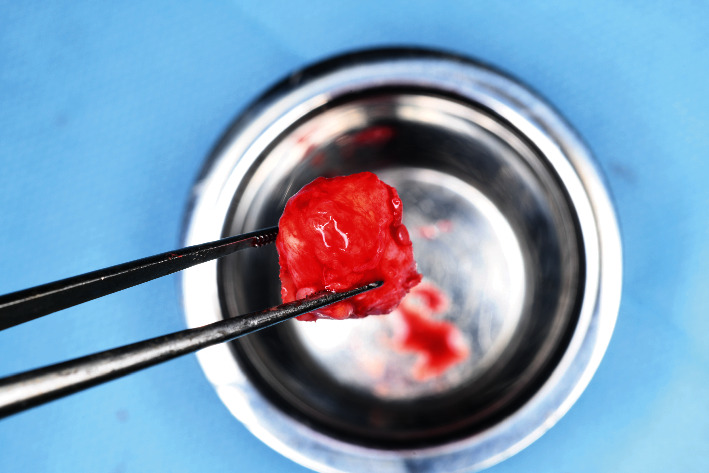
A triple graft containing a layer of soft tissue and cortical and cancellous bone.

A triple graft was harvested from the tuberosity using a straight chisel with an 8-mm scoop-like working tip (1676-08; Lexer Mini, A. Schweickhardt GmbH & Co. KG, Germany) from the Immedaite dentoalveolar reconstruction (IDR) kit developed by Dr. José Carlos Martins da Rosa (Figure 8).

**Figure 9:**
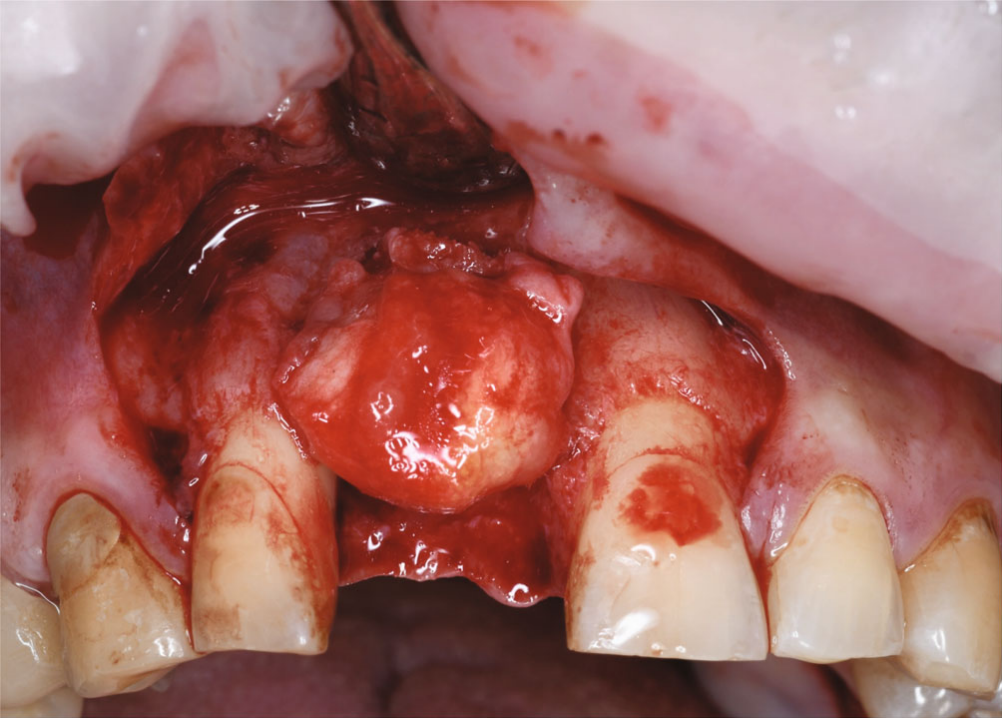
The block securely positioned into the defect.

A chisel was placed 90° to the distal wall of the socket from inside parallel to the occlusal surface of the tuber. The bone block was harvested in one piece and was slightly larger than the defect to compensate for shrinkage. The triple graft was then wedged into the apical part of the defect and was delicately compressed within the coronal portion (Figure 9). Once the graft was fitted into the defect, very tight adaptation of its cancellous layer was seen along the defect walls. In some cases, if required, rongeurs (R15 Rongeurs; Hu-Friedy Mfg. Co. LLC, Frankfurt am Main, Germany) or scissors (S14, La Grange scissors; Hu-Friedy Mfg. Co. LLC, Frankfurt am Main, Germany) were used to trim the margins of the graft while making adjustments.

**Figure 10:**
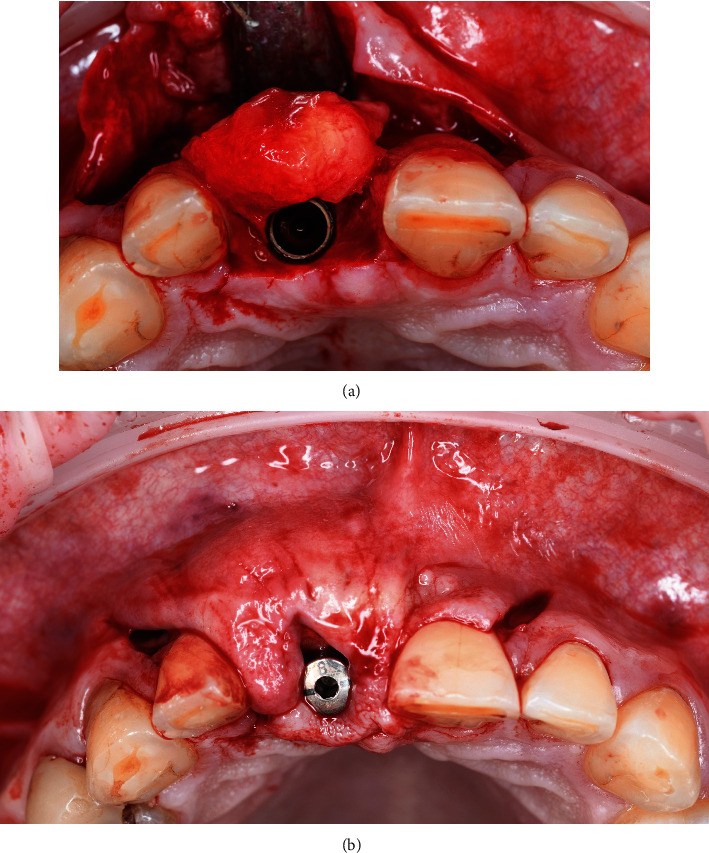
An AstraTech OsseoSpeed^™^ TX implant with a standard healing abutment.

After the bone block was well adapted to the defect, an AstraTech OsseoSpeed^™^ TX implant (Ref. #24942, AstraTech Implant System; Dentsply Sirona, Dentsply Implants Manufacturing GmbH, Hanau, Germany) with a diameter of 4.0 mm and length of 11 mm was placed with a torque of 35 N·cm. Since TX implants have parallel walls and are not designed for immediate or early implant placement, the implant bed was underprepared with sequential drilling until up to 3.35 mm into the coronal part in accordance with the surgical protocol recommended by the manufacturer. A standard prefabricated healing abutment (Ref. 24576; AstraTech Implant System, Dentsply Sirona, Dentsply Implants Manufacturing GmbH, Hanau, Germany) was placed on the implant at the time of suturing to facilitate wound closure (Figures 10(a) and 10(b)).

**Figure 11:**
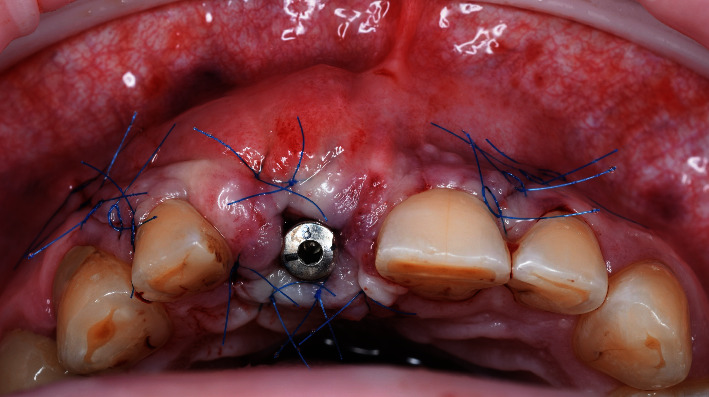
Passive wound closure.

To ensure passive wound closure, flap mobilization was performed by periosteal splitting parallel to the bone inside the buccal flap with a new 15C blade (10-256-12; Hu-Friedy Mfg. Co. LLC, Frankfurt am Main, Germany). If sufficient flap mobilization was not achieved, blunt scoring with a Castroviejo needle holder or internal flap stretching with a periodontal knife could be performed as well (KO12KPO3AR; #1/2 Allen Orban Knife; Hu-Friedy Mfg. Co. LLC, Frankfurt am Main, Germany). After the flap passively covered the augmented area, single interrupted sutures were placed palatally to ensure adaptation of the passive flap. For the second step, vertical releasing incisions were sutured from the gingival margin to the vestibulum, and additional sutures were added around the healing abutment to ensure formation of a soft-tissue seal (Figure 11).

**Figure 12:**
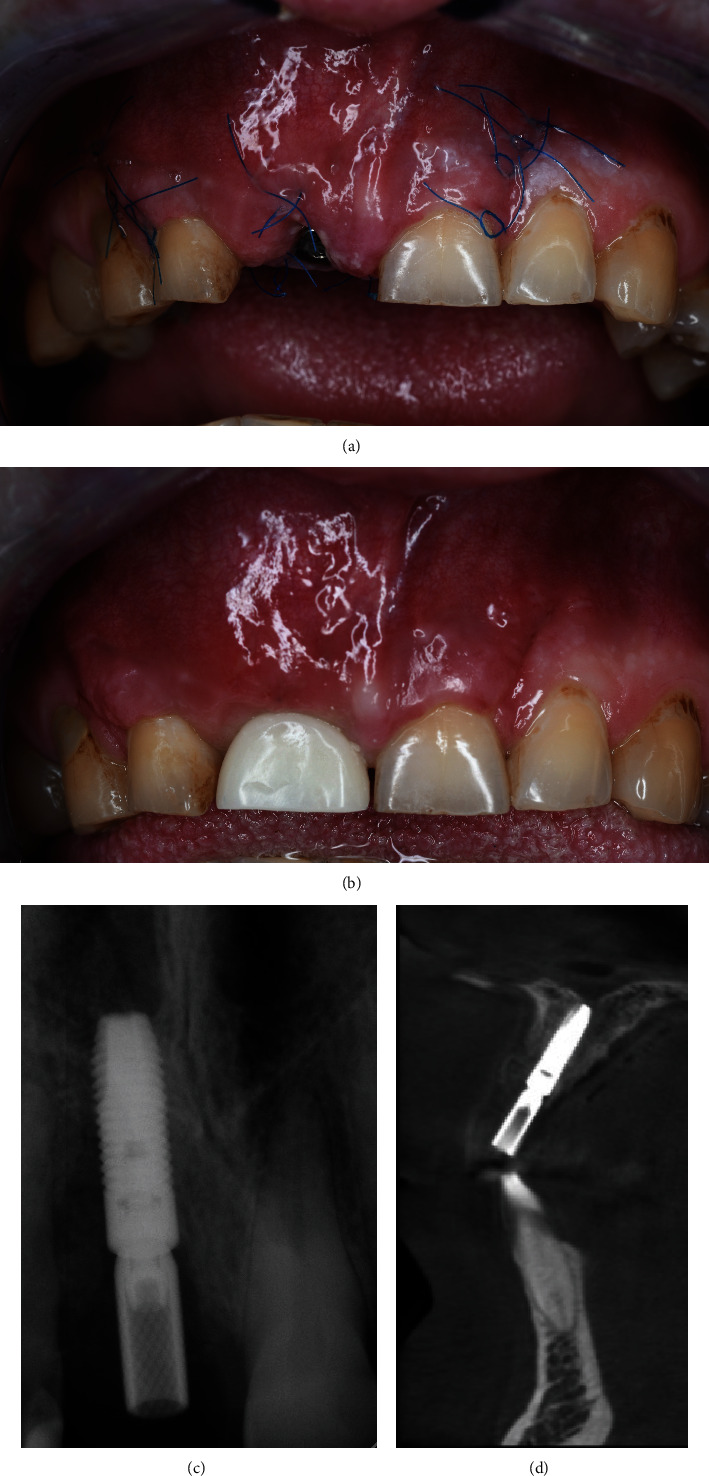
Day 5—before and after suture removal. The laboratory provisional crown is fixed.

After completing the required steps in the recipient bed, it was possible to return to the donor site. Before suturing, all the detached particles were removed. To stabilize the blood clot and to augment the lost volume after tooth extraction and graft harvesting, a collagen sponge was placed into the donor-site defect, and the wound was sutured with an X-shaped suture over the extracted third molar and with single interrupted sutures over the tuberosity. Prolene 6-0 or 7-0 (Ethicon; Johnson & Johnson MedTech, Raritan, NJ, USA) sutures were the material of choice in the majority of cases and sites. After surgery, the next appointments were scheduled daily for the next 72 hours. Sutures were removed on the fifth day, and a laboratory provisional crown was placed (Figures 12(a), 12(b), 12(c), and 12(d)).

**Figure 13:**
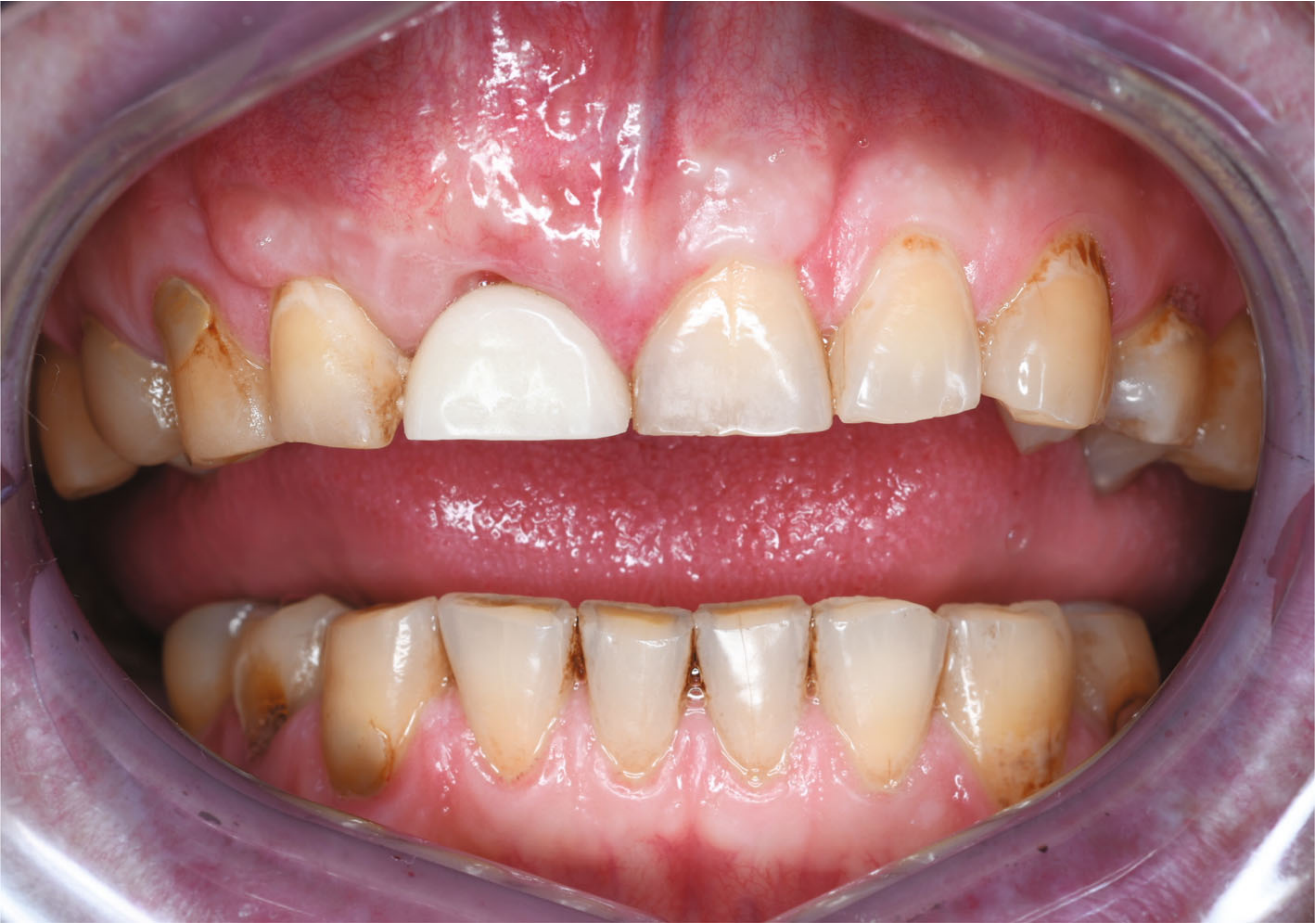
One-month follow-up.

Subsequently, the patient was followed up weekly for the first month (Figure 13) and every 3 weeks thereafter (until 3 months). Postoperative care consisted of a 0.12% chlorhexidine rinse starting 48 hours after the surgery and very soft brushing after suture removal. Since healing was uneventful (no major bleeding, swelling, or bruising was recorded), the patient did not require any painkillers or antibiotics beyond the prescribed week.

**Figure 14:**
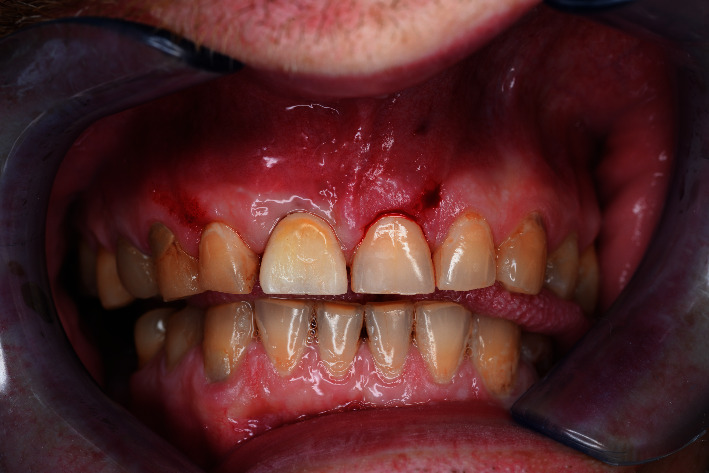
Surgical crown lengthening to level the gingival margin.

One month after the surgery, the gingival margin around the two central incisors was coronally positioned due to hard and soft-tissue augmentation and coronal advancement of the flap. Thirty-five days after the surgery, soft-tissue contouring could be started with a provisional crown placed on the implant to relocate the gingival margin apically and to accentuate the zenith. Gingivectomy of the symmetrical central incisor was performed on the same day (Figure 14). Soft-tissue conditioning with a provisional crown was performed from day 35 until the day of final restoration placement.

### 2.4. Outcomes and Follow-Up

**Figure 15:**
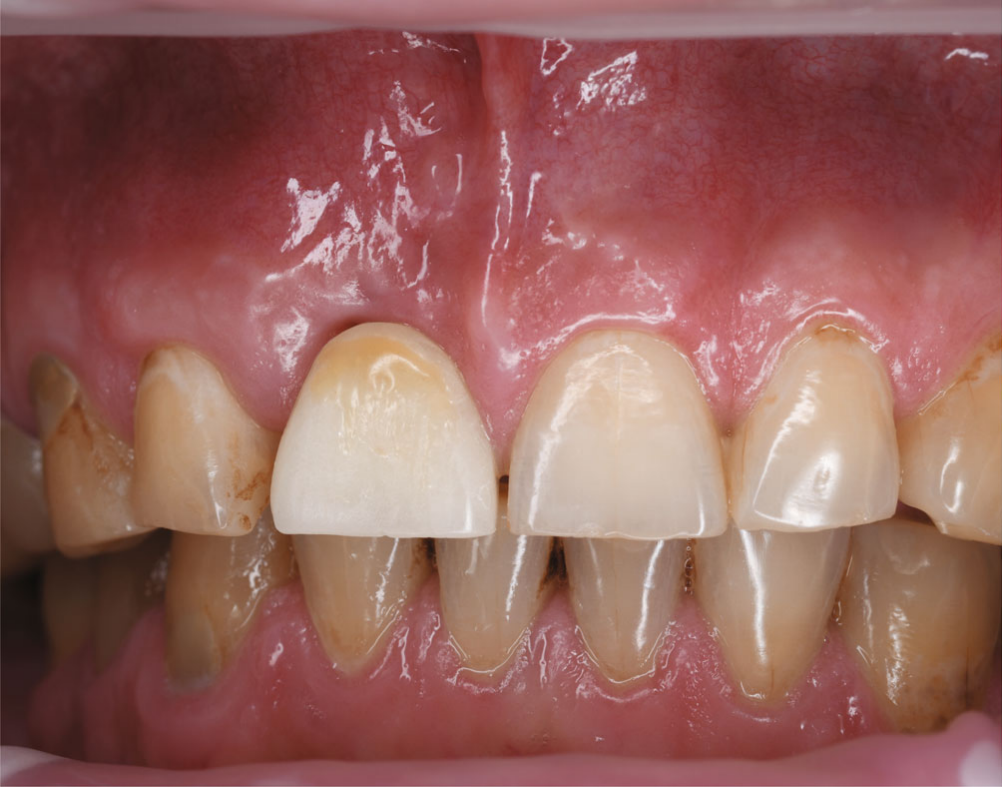
Soft-tissue maturation 2.5 months postoperatively.

**Figure 16:**
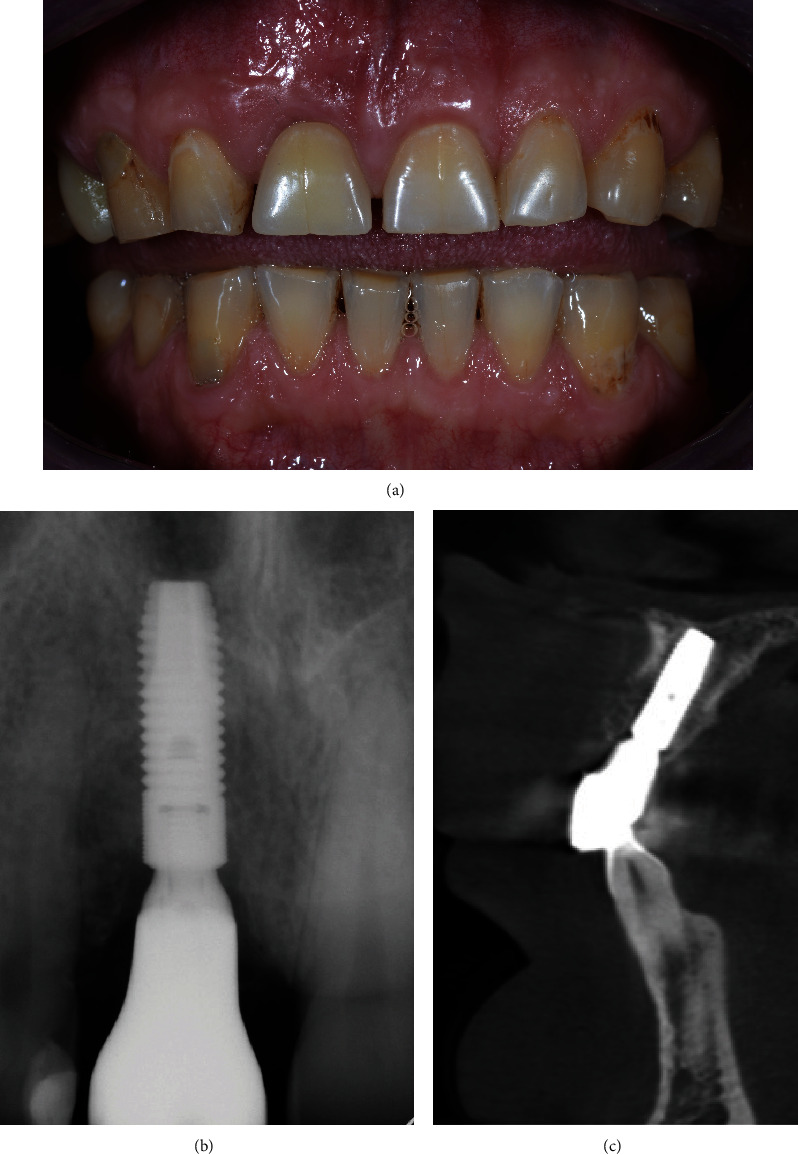
Soft-tissue stability 9 months postoperatively.

**Figure 17:**
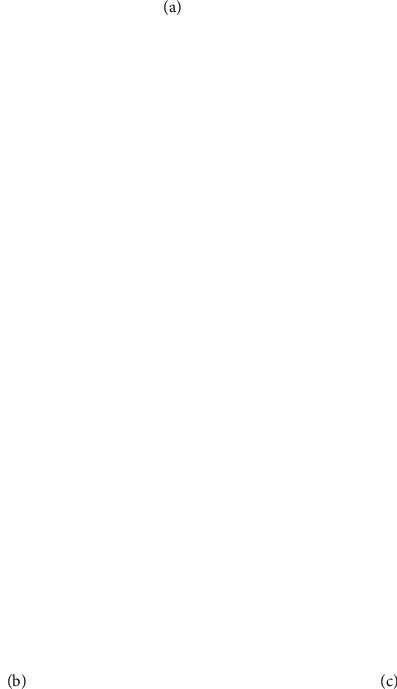
Two-year follow-up.

Despite the complexity of the initial defect, the final treatment goal could be achieved in less than 3 months owing to the restoration of the hard and soft-tissue morphology. Even with heavy smoking and poor oral hygiene, the vertical releasing incisions were not visible 2.5 months after the surgery when the final impressions were taken (Figure 15). At the same time, mucointegration was still ongoing and could be seen during tissue retraction. A definitive screw-retained crown was placed 6 months after the surgery. Follow-up examinations confirmed continuous soft-tissue maturation and keratinization. The gingival architecture and marginal bone remained stable over time (Figures 16(a), 16(b), 16(c), 17(a), 17(b), and 17(c).

## 3. Discussion

**Figure 18:**
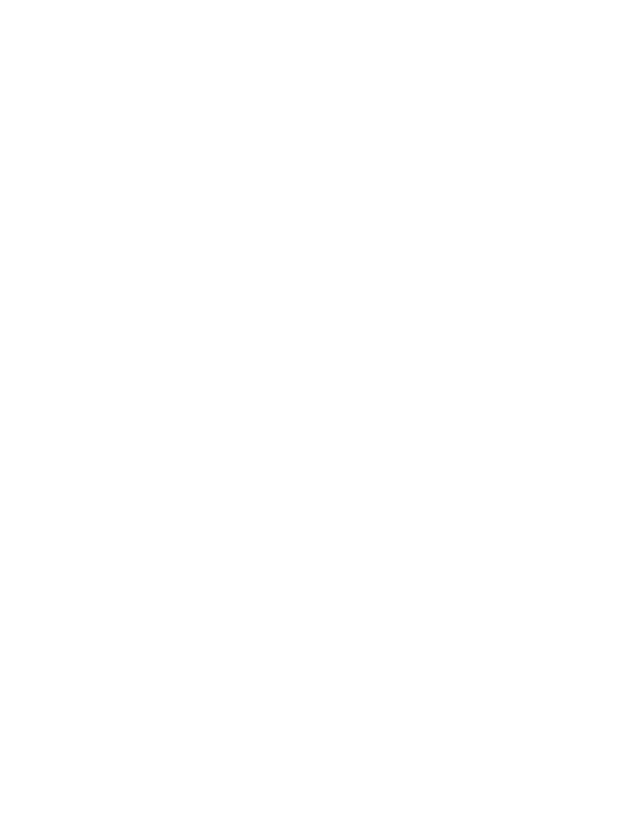
Periosteal invagination into the cancellous bone (blue, periosteum; yellow, functional periosteal layer; red, lamellar bone). Mallory's trichome stain, ×50.

Numerous bone-augmentation techniques have been described in the dental literature with a good level of evidence [11, 12]. However, very few of these techniques allow complete bone and soft-tissue reconstruction at sites with large defects within a period of 3–4 months and provide stable long-term results. In a similar clinical environment as described in this case, vertical bone-augmentation experts would recommend extraction of the lateral incisor due to the attachment loss and the missing proximal bony peak. The potential height of the vertical bone fill is usually calculated from the bony peaks mesial and distal to the defect. However, in the present case, the lateral incisor could be saved, and the lost volume of hard and soft tissue could be augmented by combining the augmentation procedure with implant placement. Nevertheless, the question of clinical attachment gain at the lateral incisor remains unanswered and requires further histological examination. We hypothesize that the reason for this extraordinary result is the choice of grafting material. Tuberosity grafts seem to have very high regeneration potential and are rich in periosteal cells, bone marrow, and pre-osteoblasts, which can be seen in a histological sample of this patient's tuberosity (Figure 18). In addition, these grafts show a very high content of cancellous bone, which has been confirmed through traumatology, thereby leading to earlier bone union than that achieved with cortical grafts [13].

The use of a tuberosity corticocancellous graft for bone augmentation was first described by José Carlos Martins da Rosa [14], although this donor site has often been used for connective tissue grafting in dental literature [15–18]. Since many successful cases involving the classical flapless IDR technique have been developed by da Rosa for compromised sockets [19, 20], evaluation of the results of the open-flap approach will be of clinical significance.

Despite the limitations of a case report, the findings of the current case indicate that clinical and radiographic marginal bone stability can be achieved within 2 years after implant surgery. An open flap does not affect the outcome; on the contrary, it provides better control of hard and soft tissues during surgery and enables the placement of a tuberosity graft with direct vision of the site. In addition, an open-flap approach allowed for the extension of the indications for this type of implant rehabilitation. Implant installation following an International Team of Implantology (ITI) type I or type II protocol [21], immediate provisionalization, and alveolar defect augmentation during a single surgical intervention offer incontestable advantages for both dental professionals and patients.

However, there is no consensus regarding immediate (type I) implant placement, since the majority of clinicians still consider that it can be indicated in 5%–20% of cases depending on the baseline characteristics, such as a thick soft-tissue phenotype, a buccal bone thickness of 1.5–2 mm, fully intact socket walls, and primary stability of at least 35 N·cm [22]. On the other hand, the appropriate choice of bone-augmentation technique and strict surgical and prosthetic protocols may enhance the success and long-term stability of immediate or early implants. A maxillary tuberosity graft may compensate for volumetric changes and marginal bone reduction post-extraction if augmentation is combined with immediate or early implant placement, which can be another influencing factor.

Although ridge alteration post-extraction is inevitable due to the loss of bundle bone [23, 24], it may yield promising results with type I or type II implant rehabilitation simultaneous with bone augmentation thanks to the tuberosity tissue quality and composition, as well as to the right choice of implant design. However, the efficacy of the B2S approach has been proven only with 18 years of clinical experience of one of the authors and requires more clinical and scientific validation.

## 4. Conclusion

This case presents a novel approach for combined alveolar defect reconstruction and implant installation that can be successfully performed using an autogenous graft harvested from the maxillary tuberosity. The tuberosity has been well-described as a donor site for connective tissue grafts in the literature, but the regeneration potential of corticocancellous grafts should be better investigated in comparative studies, which rank higher in the scientific hierarchy. The open-flap (B2S) approach provides more freedom during surgery and can demonstrate stable results postoperatively. It also does not require strict patient selection, thereby extending the indications for immediate reconstruction of dentoalveolar defects.

## Data Availability

The [Data Type] data used to support the findings of this study are included within the article.
